# The Prevalence of Accidental Needle Stick Injury and their Reporting among Healthcare Workers in Orthopaedic Wards in General Hospital Melaka, Malaysia

**DOI:** 10.5704/MOJ.1407.009

**Published:** 2014-07

**Authors:** A Bhardwaj, N Sivapathasundaram, MF Yusof, AH Minghat, KMM Swe, NK Sinha

**Affiliations:** Department of Orthopaedics, Newcastle University Medicine Malaysia, Johor, Malaysia; Department of Orthopaedics, Melaka General Hospital, Melaka, Malaysia; Department of Orthopaedics, Melaka General Hospital, Melaka, Malaysia; Department of Orthopaedics, Melaka Manipal Medical College, Malaysia; Department of Orthopaedics, Newcastle University Medicine Malaysia, Johor, Malaysia; Department of Orthopaedics, Melaka Manipal Medical College, Malaysia

## Abstract

**Background :**Accidental needle-stick injuries (NSIs) are a hazard for health-care workers and general public health. Orthopaedic surgeons may be more prone to NSIs due to the prevalence of bone spikes in the operative field and the use of sharp orthopaedic instruments such as drills, saws and wires. A hospital-based cross sectional study was conducted in the orthopedic wards of Melaka General Hospital. The prevalence of NSIs was 32 (20.9%) and majority of it occurred during assisting in operation theatre 13(37.4%). Among them six (18.8%) were specialist, 12(37.5%) medical officer, 10 (31.2%) house officer and four staff nurses (12.5%). Among the respondents 142 (92.8%) had been immunized against Hepatitis B and 148 (96.7%) participants had knowledge regarding universal precaution. The incidence of NSI among health care workers at orthopaedics ward was not any higher in comparison with the similar studies and it was found out that the prevalence was more in junior doctors compared with specialist and staff nurses and it was statistically significant.

**Key Words:**

Needle sticks injury, health care workers, and standard precaution

## Introduction

Needle stick injuries (NSIs) are the most common occupational hazard that a health care worker (HCW) is exposed to in the work place. The Ministry of Health Malaysia defines needle stick injury as injury caused by suture or hollow-bore needles or any sharp in struments ^1^ . The occupational Health Unit in the Ministry of Health, Malaysia, reported an incidence rate of 4.7 needle stick injuries per 1,000 HCW’s in 2005^2^ . According to the World Health Organization (WHO) data, 35.7 million health care workers in the world are exposed to the risk of NSIs ^3^ .

The Health Protection Agency (HPA) report in 2012 on health care workers stated that injuries during occupational exposure among the medical and dental professions increased by 131% (100-231) from 2002 to 2011. Majority of these occurred among the ancillary staff. These exposures were attributed to non compliance with standard infection control precautions for the handling and safe disposal of clinical waste ^4^ .

The potential effects related to these injuries include risk of transmission of blood borne pathogens such as human immunodeficiency virus (HIV), hepatitis B virus, (HBV) and hepatitis C virus (HCV). In a WHO study, the annual global estimated proportion of health care workers exposed to these infections were 0.5% for HIV, 2.6% for HBV and 5.95% for HCV 3. The total number of HCV seroconversions reported between 1997-2011 is 20 (17 from England and three from Scotland). Kubitschke A et al stated that the overall risk of acquiring an HCV infection after a needle stick injury was lower than frequently reported. The risk of acquiring acute HCV infection was lower in Europe (0.42%) in comparison to Eastern Asia (1.5%) where an HCV viraemia was reported during follow-up. There is considerable worldwide difference in HCV seroconversion rates probably related to genetic factors that may provide some level of natural resistance against HCV.^5^ According to the HPA report the last case of an HIV seroconversion was reported in 1999 ^4^.

Besides infections, the long term outcome of health care workers who sustained needle stick injury include substantial psychiatric morbidity such as depression, including post-traumatic stress disorder (PTSD) and adjustment disorder (AD). The attendant consequences to these effects include missed work days which directly affects the health care services and resources ^6-7^.

Orthopedic surgeons are more prone to needle stick injuries due to exposure to bone spikes in the operative field and the use of sharp orthopedic instruments such as drills, saws, kirschner wires and pins. It is reported that an orthopedics surgeon’s risk of sharp injury is as high as 80%-90% in a 10-year period.^8^ The mean exposure rate among the orthopaedic surgeons is 1.4 per year with only 33 % reporting the incident. The reason for not reporting was identified as a perceived low risk of the patient carrying the blood borne infections and possible stigmatization and loss of employment ^9-10^.

The objectives of this study are to determine the prevalence of accidental needle stick injuries (NSIs) among the health care workers in the department of orthopaedics, clinical situations, their responses after a needle stick injury episode and to determine their level of knowledge of blood borne pathogens and practice of standard work precautions.

## Materials and Methods

This is a cross sectional study conducted in Melaka General Hospital from February 2013 to March 2013 in the department of orthopaedics.

Sample size was calculated by using Epi info with 95% confidence interval and prevalence rate of 23% ^17^. All the health care workers posted at Orthopaedic Ward, Melaka Hospital participated in this study. The questionnaires were developed based on the health belief model.^26^ Data was collected in the form of pre-tested self-administered questionnaires. In order to standardize the questionnaires, a pilot study was conducted among house officers and content validity was examined.

After the institutional review board and MREC approval, a structured questionnaire was used to gain information on socio-demographic profile of the respondents, history of needle stick injuries, types of devices causing the injury, their reporting and knowledge on standard precautions, post exposure prophylaxis and their risk perception on needle stick injuries. The questionnaire was translated into Bahasa Malaysia and the questionnaires were administrated with face to face interviews to generate adequate response rate. Needle stick injury was defined as percutaneous injury caused by the suturing needle and hollow bore needles or sharp instruments, that is, the type of needle used for giving injection or drawing of blood which has the bore that the blood can remain inside after use. Cases of needle stick injuries were respondents who had one or more experiences of needle stick injury.

Data collected were analysed using with SPSS software version and chi square test and were used to determine factors associated with needle stick. The statistical analysis was conducted with 95% confidence interval and a p-value of <0.05 as threshold of statistical significance.

## Results

**I. Baseline characteristics of respondents**There was total of 153 health care workers who participated in this study, which included 85 doctors (10 specialist, 25 medical officers and 50 house officers) and 68 staff nurses. Among the respondent, 62 (40.5%) were male and 91 (59.5%) were female. The majority of the respondents were Malay 107 (69.9%). The mean age of respondents was 31 years (SD, 7.49) (range 22 to 57 years). It was observed that 142 (92.8%) respondents were immunized with Hepatitis B vaccine and 11 (7.2%) were not immunized at all. In this study 148 (96.7%) participants had knowledge on standard precautions.

**II. Prevalence of needle sticks injury**There was a total of 32 (20.9%) reported episodes of needle stick injuries in this study. Among them 18 (56.25%) were male and 14 (43.75%) female health care workers (P < 0.034).

**Fig. 3: Categories of health staff that were injured.**
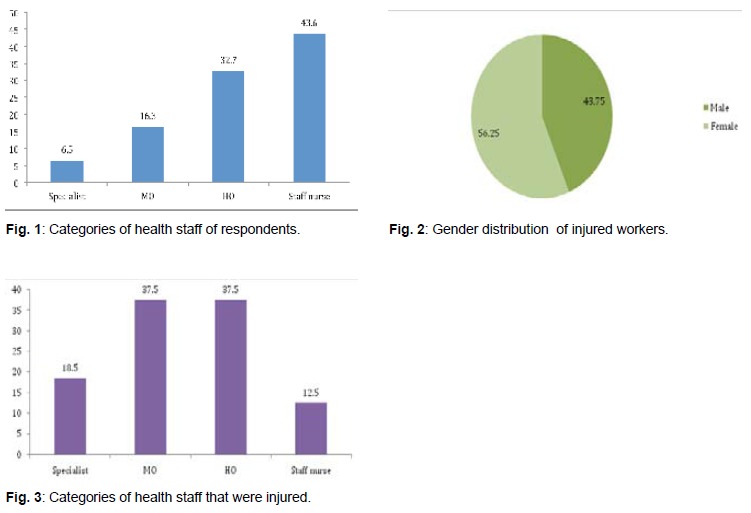


Majority of injuries occurred while assisting surgery in the operation theatre 13 (37.4%). Amongst them 6 (18.8%) were specialists, 12 (37.5%) medical officers, 10 (31.2%) house officers and four (12.5%) staff nurses. (Figure 3) Majority of injuries were due to hollow bore needle 15 (46.8%) and 16 (50%) due to solid needle and one (3.2%) due to bone spike. Twenty three (71.8%) of the injuries were self-inflicted and among them 31 (96.9%) were wearing gloves during the injury.

Site of injury revealed that 15 (47%) of respondents had injuries in a finger and 17 (53%) had injury in the hand. Factors were explored for perceived cause of injury and the majority 12 (37.5%) of injuries had occurred when the procedure was hurried, 5 (15.6%) due to tiredness, 5 (15.6%) due to lack of experience and others due to poor lighting Twenty-seven (84.4 %) of the workers had taken immediate post incident action but five (15.6%) had not taken any appropriate action. The immediate post-exposure action taken was washing the injured part 14 (51.8%), medication 6 (23.1%), and two others (7.4%) such as informing senior staff. Majority of those who did not take any immediate action perceived that there was no need to take any action.

**III. Knowledge concerning needle stick injury**Knowledge of risk of needle stick injury and standard precautions were assessed and the results of correct answers are in Table (I). Most of the respondents had knowledge on the diseases transmitted by contaminated sharp objects.

Regarding knowledge score 67.3% of respondents had good knowledge and only 32.7% had fair knowledge which needed to be improved through training and health education Table (II).

**IV. Perception of students on needle sticks injury Risk perception of needle stick injury**Ten questions were designed to assess the perception of health care workers on risk of needle stick injury and the results are shown in table (IV). Regarding perception score of the respondents, 111 (72%) demonstrated high positive perception on needle stick injuries Table (III).

## Discussion

NSIs are a potentially serious threat to health care workers exposing them to the risk of acquiring blood borne pathogens through sharps or instruments. The prevalence of needle stick injuries in our study is 20.9%.It is lower compared to prevalence of 24.9% reported by Lee and Hassim in 2005, 23.5% Rampal et al 2010 ^11,12^. In a study by Ng and Hassim in 2003 in the accident and emergency department of two teaching hospitals prevalence rates was 31.6% and 52.9% ^13^. This may be related to ‘selection bias’ as the study groups were selected form high risk backgrounds such as accident and emergency departments. The studies from Pakistan and India are consistent with higher prevalence rates ^14^. These variations can be attributed to the existence of stringent infection control program, regular training of the staff on the standard precautions, inclusion of infection control practices in the teaching curriculum of the medical students, nursing staff, surveillance and the regular practices of use of gloves and availability of safety devices.

In our study the male respondents reported a higher prevalence although most studies did not show any sex difference. The concerns are for the females in the reproductive age group and their future pregnancies compromised due to blood borne infections. Ninety-three percent (93%) of the health care workers in our study reported HBV immunisation. The concern is in the 7% of respondents who had not completed the immunisation schedule placing the health care worker at risk of infection due to inadequate antibody response. According to Lee and Hassim 2005 these workers may also suffer from a false sense of security and may not seek post exposure prophylaxis ^9^. It is estimated that 40% of hepatitis B-, and C and 2.5% of HIV are attributed to needle stick injuries ^11^. In a study by Wallis et al on the perceptions of orthopaedic surgeons on hepatitis C transmission, it was identified that they were mostly unaware of the true prevalence of this infection especially in high risk groups such as intravenous drug abusers. The author concluded that there was a need for greater awareness of all aspects of hepatitis C infection and its associated risk in the practice of surgery ^9^.

The devices responsible for needle stick injuries in our study were solid suture needles in orthopaedic surgeries (46.9%), followed by hollow bore needle (46.9%), and bone spikes (3%). In terms of number of episodes based on the recall of the respondents about 11.8% reported one episode over the past two years and junior doctors (training medical officers and house officers) reported highest prevalence in our study. Solid bore needles were most often used in suturing and the fact that the junior doctors reporting injury explained the increased likelihood of injury in earlier stages of training. The selfinflicted injuries were commonly noted during surgeries while transferring the sharps between the personnel or discarded needles in the tray /kidney dishes and among the drapes. The alternate viewpoint is the likelihood of junior doctors injuring themselves in wound closure at the conclusion of surgery, whereas the senior doctors are more at risk of exposure to device injuries during surgery. In a nationwide survey among Danish physicians on percutaneous exposure, it was observed that suture needles were primarily responsible for the injuries, followed by intravenous catheters, injection needles, scalpels and bone fragments ^15^.

Although there is a lack of extensive studies in evaluating sharp injuries in orthopaedic surgery, there are studies that demonstrated glove perforation rates of 15.8% in major and minor orthopaedic procedures and the right thumb and left index finger having the maximum injuries ^16^, consistent with our study where 10.5% of injury were reported on the fingers.

There are studies demonstrating highest incidence of needle stick injuries among nurses as injections were mainly administered by them and were frequently involved in drawing blood. The other frequent episode of needle stick injury was during intravenous cannulation and venepuncture with hollow bore needle. In a study by Jagger et al, it is reported that one third of injuries occurred during recapping of the needle ^17^. The recapping of needle is strictly prohibited under the Occupation Safety and Health Administration (OSHA) blood borne pathogen standard ^18^. Our study demonstrated that 92% of respondents had knowledge that recapping of needle is an unsafe practice.

The other category of personnel at high risk include the medical students as shown by Norsayani et al and Bernard et al related to their natural enthusiasm of learning new technical skills ^19,20^. There are currently wide availability of safety engineered needles and sharps. These new generation devices have demonstrated enhanced safety in performing procedures associated with high risk of blood borne transmission of pathogens, such as intravenous cannulation and drawing blood. Two studies have reported 74% to 89% reduction in the needle stick injuries with the usage of safety engineered devices ^21, 22^.

A study investigated the orthopaedic uses of safety engineered devices, on outpatient arthrocentesis, intraarticular injections, aspiration biopsy, and ultrasound guided procedures. The observation identified no accidental needle sticks (0 needle sticks per 1300 devices). The author concluded that the shielded safety needles, mechanical syringes, manual retractable syringes, and shielded syringes, are effective, reliable and safe for orthopaedic syringe procedures ^8^.

Further exploration of the reason for the needle stick injury in our study identified hurrying to complete the task, fatigability and lack of experience on safe handling of devices. Mental and physical stress related to long working hours is documented to increase the risk of injuries as it impedes the practice of safe procedures ^23^. This is consistent with our findings in which 42 % of respondents were of the opinion that long working hours were positively associated with occurrence of injuries. Extended work injuries occurred after a mean of 29.1 of consecutive work hours, non-extended work injuries occurred after a mean of 6.1 consecutive work hours. A study among the nurses reported a statistically significant increase in needle stick injuries with working for more than 8 hours per day (P < 0.05) ^24,25^.

Hence the need for greater reinforcement on scheduled day off and opportunity to sleep seemed important. A study by Patricia et al on shift level staffing association with needle stick injury, identified that episodes occurred even in the presence of shift and it was the lower staffing during the shift with increased patient-to-nurse ratios that correlated with elevated risk for injury ^26^. Hence it has implications for policy makers and hospital administrators to ensure adequate staffing that is a potential problem in developing countries.

About fifteen percent (15%) of respondents in our study did not take any post-exposure measures with the fear of blame, perceived low risk of infection and exposure and lack of knowledge of how and to whom to report. Medical students were often recognised as likely to ignore reporting as evidenced in few other studies ^11,22^. The reason for this being the lack of clear guidelines on the reporting process and Bernard et al stated that it was because of their concerns of negative effect on their evaluation if they were to interrupt a case in the pursuit of reporting the exposure ^20^. The Ministry of Health in Malaysia-, envisages that all cases of needle stick injuries be reported within 24 hours to the infection control team of the hospital or the head of department or to the safety and health committee ^27^. Although this is the policy, reporting is purely voluntary and hence the overall prevalence of the cases remain uncertain and the magnitude of the problem cannot be accurately determined.

This study shows that although majority of respondents (67.3%) demonstrated awareness about the universal precaution guidelines, and exhibited high risk perception (72.5%) however, there is scope for improvement (32%) in the knowledge gaps on the precaution guidelines. There was no difference in median score of knowledge on standard precautions among the cases of needle stick injuries and other non-needle stick injuries. We believe that reiteration about NSI and analysis of present practices should help to enhance awareness and to reduce the incidence of NSI. The fact that needs reiteration besides knowledge is the practice of standard precautions that will prevent the health care workers from risk of needle stick injuries and accompanied by adequate reporting that will benefit them through timely interventions.

The Centre of Disease Control (CDC) reiterates the importance of a culture of safety in work place to prevent occupational hazards. The strategies that should be employed to change staff practices towards safety measures include establishing multidisciplinary injury-prevention teams which includes representatives from all disciplines at risk for exposure, written exposure control plan, with a hard copy available to all employees, enforcing sharps injury reporting and records and educating front line workers. Such a training should be undertaken at the time of employment and further more once annually ^28^.

The limitation in our study is the response rate with a possibility of underreporting. It is documented that self-reporting of needle stick injuries do not capture all the occurrences due to recall inaccuracy ^29^. In a study by Lee et al, passive surveillance reports detect only a small percentage of needle stick injuries compared to reports in prospective studies ^30^. To fully understand the exact magnitude of the problem, a multi-centric study is required. However our present study provides the provisional report of the prevalence of the needle stick injuries among the orthopaedic surgeons and the attending nurses. It also highlights those gaps in the knowledge and practices on standard precautions and safety of devices that need to be addressed. Furthermore, under-reporting of the incidents do occur and there are opportunities to facilitate a climate of ensuring safety of all the health care workers and improve patient care ^31^.

**Table I: Knowledge of NSI by respondents.**
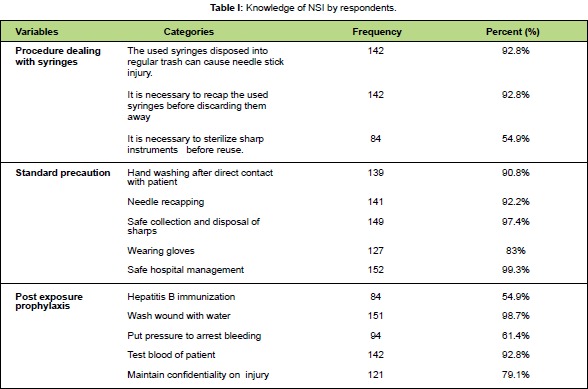


**Table II: Knowledge score of the respondents:**
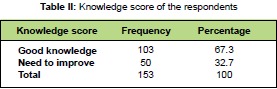


**Table III: Perception score of the respondents:**
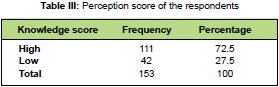


**Table IV: Questionnaire on Perception of needle stick injury:**
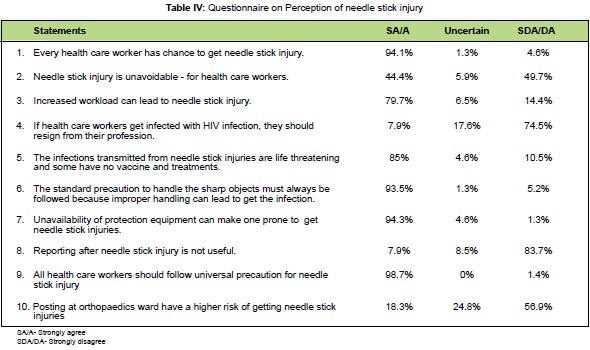


## Conclusion

In conclusion, this study reveals that health care workers in the orthopaedic department are at risk of needle stick injuries through solid needles during surgical procedures and hollow needles while drawing blood and intravenous cannulation. The concerns that require further emphasis include non-adherence to pre and post exposure precautions, under-reporting of the exposures by the staff that mandates additional research to elaborately explore the factors that suppress the practice and reporting. There is a need to reiterate on completing the three doses of Hepatitis B immunisation. The prevalence is high among the junior doctors and the nurses. Although there is adequate demonstration of knowledge on standard precautions, it is not fully practised. Practice of standard precautions for infection control needs to be followed by all health care workers irrespective of the patient diagnosis and perceived infection status. Training and education on workplace safety, safe handling and disposal of sharps, provision of personal protective equipment, availability of safety engineered devices, IEC materials displaying the protocol and flow chart of reporting on exposures and access to interventions are the various ways to prevent this preventable occupational hazard.
